# Direct 3D Printing of Clear Orthodontic Aligners: Current State and Future Possibilities

**DOI:** 10.3390/ma14071799

**Published:** 2021-04-05

**Authors:** Gianluca M. Tartaglia, Andrea Mapelli, Cinzia Maspero, Tommaso Santaniello, Marco Serafin, Marco Farronato, Alberto Caprioglio

**Affiliations:** 1 Department of Biomedical, Surgical and Dental Sciences, School of Dentistry, University of Milan, 20100 Milan, Italy; gianluca.tartaglia@unimi.it (G.M.T.); andrea.mapelli@3dobj.ch (A.M.); cinzia.maspero@unimi.it (C.M.); marco.farronato@unimi.it (M.F.); alberto.caprioglio@unimi.it (A.C.); 2 Fondazione IRCCS Cà Granda, Ospedale Maggiore Policlinico, 20100 Milan, Italy; 3 Department of Physics, University of Milan, 20100 Milan, Italy; tommaso.santaniello@unimi.it

**Keywords:** 3D printing, clear aligners, dental printing resin, malocclusion, narrative review, orthodontics

## Abstract

The recent introduction of three-dimensional (3D) printing is revolutionizing dentistry and is even being applied to orthodontic treatment of malocclusion. Clear, personalized, removable aligners are a suitable alternative to conventional orthodontic appliances, offering a more comfortable and efficient solution for patients. Including improved oral hygiene and aesthetics during treatment. Contemporarily, clear aligners are produced by a thermoforming process using various types of thermoplastic materials. The thermoforming procedure alters the properties of the material, and the intraoral environment further modifies the properties of a clear aligner, affecting overall performance of the material. Direct 3D printing offers the creation of highly precise clear aligners with soft edges, digitally designed and identically reproduced for an entire set of treatment aligners; offering a better fit, higher efficacy, and reproducibility. Despite the known benefits of 3D printing and the popularity of its dental applications, very limited technical and clinical data are available in the literature about directly printed clear aligners. The present article discusses the advantages of 3D printed aligners in comparison to thermoformed ones, describes the current state of the art, including a discussion of the possible road blocks that exist such as a current lack of approved and marketed materials and limited existence of aligner specific software. The present review suggests the suitability of 3D direct printed aligners is superior to that of thermoformed manufactured aligners because of the prior’s increased accuracy, load resistance, and lower deformation. It is an overall more stable way to produce an aligner where submillimeter movements can make a difference in treatment outcome. Direct 3D printing represents a complex method to control the thickness of the aligner and therefore has a better ability to control the force vectors that are used to produce tooth movement. There is currently no other approved material on the market that can do this. The conclusion of this article is that we encourage further in vitro and in vivo studies to test these new technologies and materials.

## 1. Background

### Brief History

**Figure 1:**
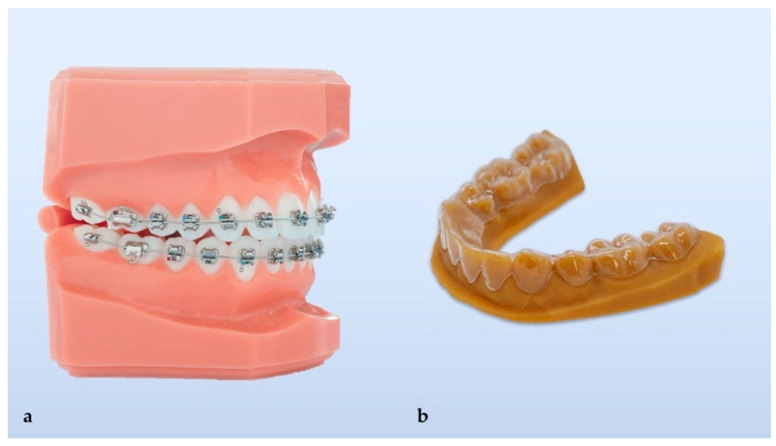
Conventional orthodontic treatment (**a**) and thermoformed clear aligner with its 3D printed mold (**b**).

Orthodontics is now approaching its fourth revolution since its inception as a specialty of dentistry in the early 1900s. In those days, malocclusion was treated with the application of metal rings cemented to teeth to support wires for applying moving forces. This initial treatment strategy for the correction of dental and skeletal malocclusion was often accompanied by a huge number of dental caries because it was almost impossible to maintain correct dental hygiene due to the limited offering of dental hygiene tools in the market at that time and to the mechanical encumbrance of the cemented rings and subsequent dental plaque formation on the teeth. Later in the 1960s, the rings were substituted by the first brackets to support wires made from stainless steel, which offered several advantages, such as high strength, durability, reduced friction, improved salivary flow, less food collection, and they were relatively easy to form. This wonderful technological jump represented a revolution and was made possible due to the development of dental science materials able to bond the brackets directly onto the teeth. The conventional brackets made of transparent or translucent non-metallic material were introduced in the early 1970s (Figure 1). The first transparent brackets made of unfilled polycarbonate were later replaced by ceramic-reinforced, fiberglass-reinforced, and polycarbonate-reinforced brackets with a metallic insert to minimize creep deformation [1,2]. Later, ceramic brackets (monocrystalline sapphire and polycrystalline ceramic) were used; however, the hardness of those materials could cause enamel abrasion [3].

Similarly to advances in material used for brackets, there has been a technological progression in the different types of archwire have been used since the popularization of orthodontic treatment. Initial use of traditional stainless steel archwire was eventually replaced by clear optical fiber (Optiflex), Teflon coated wire, epoxy coated wire, titanium plastic coated wire and Bioforce wire [3]. This technological development led to the third revolution and represents current contemporary orthodontic science.

Towards the end of the last century and in the first decade of this century, the first aligners for orthodontic purposes appeared [4]. This revolutionary development was mainly driven by the pressure of patients seeking more comfortable and less intrusive orthodontic procedures for their teeth. Indeed, since their introduction as a treatment option in orthodontics, aligners have enjoyed great popularity and the number of patients undergoing orthodontic treatment with aligners has only increased. The use of aligners in orthodontics has created a paradigm shift for dentistry and has encouraged people of all ages, including older adults, to seek treatment for their malocclusion.

The aim of the present narrative review is to review the history of clear aligners’ manufacturing processes from thermoforming to 3D direct printing; to discuss the advantages of 3D printed aligners in comparison to previous technologies, explore the state of the art, include possible points of criticism such as a current lack of approved and marketed materials and limited existence of software packages and suggest where we need to go from the authors’ perspective. Lastly, special attention will be paid to the potential cytotoxicity of printable resins and it lays the foundation for future perspectives and options.

## 2. Main Text

### 2.1. Development of Clear Aligners- Thermoforming

Various forms of malocclusion compromise dental aesthetics and a pleasing smile and also negatively impact dental hygiene and periodontal health. There are many reasons patients seek orthodontic treatment including; medical/dental reasons, the improvement of their appearance and to boost self-esteem and confidence [3,5]. A growing number of patients and doctors are choosing orthodontic treatment with aligners, which is not only effective but also does not compromise the quality of their daily life. This is in comparison to conventional orthodontic treatment with fixed orthodontic appliances, such as orthodontic brackets and an archwire used to apply corrective forces to move the teeth into the desired position. These appliances create lip discomfort and have an unpleasant appearance [6].

The disadvantages of conventional treatment and the increasing desire of patients for alternative “invisible” treatments have contributed to the popularization of transparent aligners used in series, that is, a set of tooth positioners fabricated based on precise impressions or digital scans of the patient’s teeth, each producing different forces to progressively reach the planned dental alignment [7]. The primary benefits are clear transparent appearance of the devices and the ability to take them out when eating and while maintaining of oral hygiene, together with higher comfort and simplicity of use [8]. The treatment with clear aligners is shorter in duration and takes less chair-side time in comparison to conventional fixed braces and archwires [9].

The first clear aligners were developed as a tool for final stages of orthodontic treatment or to treat minor irregularities of tooth position only [10]. Development in the field of clear aligners eventually led to their use for the treatment of moderate to severe malocclusions. Today, many different brands of clear aligners exist, e.g., Simplifive™, Clear aligner™, Clear path™, Smilelign, MTM Clear-Aligner™, Nimrodental Clear aligner™, Clear Image Aligners™, ClearAligner™, ClearCorrect™, Nuvola^®^, Fantasmino^®^ or Invisalign™ [11,12]. The evolution of clear aligner orthodontic protocols was done using materials available in the market, and unfortunately there has been limited developmental work to introduce new materials dedicated for this purpose.

### 2.2. Materials for Thermoformed Aligners

Various thermoplastic materials, or combinations of materials, are being used for fabrication due to their excellent characteristics. These include; polyvinyl chloride, polyurethane, polyethylene terephthalate, and polyethylene terephthalate glycol [12,13]. Currently, the work flow for creating a set of clear aligners starts with virtual planning software using the initial plaster impression (which is scanned in 3D) or direct digital 3D intraoral scan of the dentition. A physical 3D model is needed for each individual aligner of the treatment set, and it is made using 3D printing, stereolithography or material jetting. Next, aligners are then fabricated by molding the clear material over the 3D model of the patient’s teeth (thermoforming or vacuum forming) and finally, trimmed. The process is long, labor intensive, and costly [14,15]. Furthermore, to date, the environmental impact of printable resins used for 3D models generated during the thermoplastic process is not well documented. Several points need to be considered including; energy consumption, waste material, and environmental pollution [16]. Regarding dentistry, especially for orthodontics, waste material is one of the main problems that need to be solved. Excess waste material produced using current techniques has significant negative environmental effects. One solution to this problem could be incorporating the use of recycled materials in 3D printers to increase the sustainability of 3D printing technology.

One of the negative results of the thermoplastic process that has been reported is that there are significant changes in the material properties in response to the heat generation used to form the material around the teeth. Ryu et al. [17] studied changes in four types of thermoforming materials after the thermoforming process. The study showed that thermoforming affects the transparency of thicker material decreasing it, and increases water absorption properties, water solubility and can also modify the surface hardness of some plastics. Previous research also suggests that the thermoforming process decreases the thickness of the aligners compared to the original dimension of the thermoplastic foil [18]. The mechanical behavior of thermoplastic material used for the production of clear aligners plays a critical role in obtaining desired clinical results in difficult orthodontic movements, along with aspects such as aligner thickness regularity and geometry design [19]. It has been observed that thermoformed clear aligners can have different thicknesses, ranging from 0.5 to 1.5 mm, which can certainly affect their properties and clinical performances while inducing dental movement by pressure on the tooth’s surface. While a higher thickness increases the delivered force by a bigger stiffness and the flexural modulus, the thermoforming process decreases these properties, depending on the thermoplastic material used and the thermoforming process performed. The homogeneity of thickness of the aligner plays an important role in the magnitude of exerted forces: discrepancies in the thickness influence the accuracy and the adaptation to the teeth [14]. Furthermore, the amount of activation and the frequency of appliance removal are factors that should be considered in thermoforming of clear aligners; Skaik et al. carried out a study with pressure sensors to confirm the efficiency of different materials used for clear aligners and they concluded that the mechanical features of the plastic polymer used, the daily removal frequency, and the magnitude of teeth activation are factors that mostly influence the generated forces [20].

Moreover, other studies showed that thermoplastic-made aligners are reactive to the intraoral environment during their use. Body temperature, humidity of oral cavity and salivary enzymes affect the aligner intrinsically, modifying its original dimension and mechanical properties [21,22]. It has been shown that after storage in artificial saliva, the elastic modulus and tensile yield stress were modified, generally reducing the mechanical properties of the polymers investigated [19]. These alterations on the aligner structure caused by the thermoforming process and intraoral environment probably influence the clinical efficacy; the modify in mechanical properties can lead to a side effects like the potential loss of efficacy due to the presence of undesired orthodontic forces acting on teeth and their attachments.

**Figure 2:**
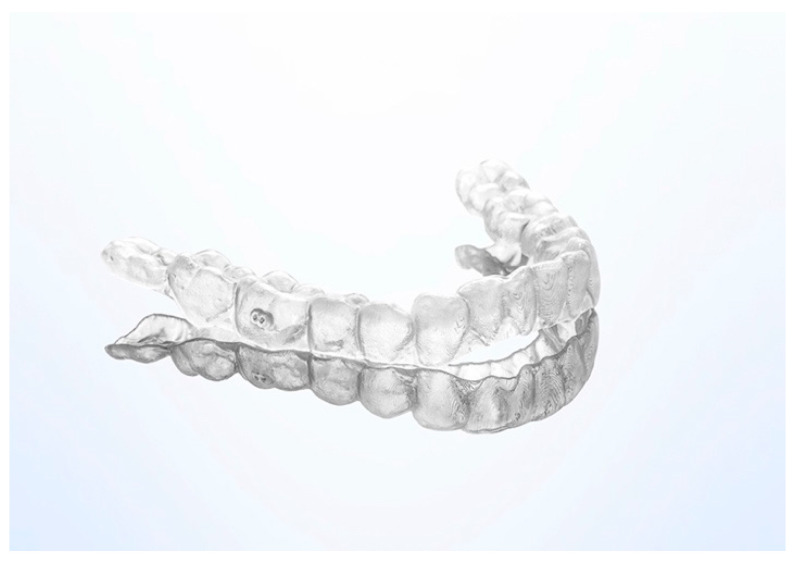
Direct 3D printed clear aligner.

In vitro cytotoxicity of human primary gingival fibroblasts of four thermoplastic materials was assessed after 14 days of exposure, namely Duran (Scheu-Dental GmbH, Iserlohn, Germany), Biolon (Dreve Dentamid GmbH, Unna, Germany), Zendura (Bay Materials LLC, Fremont, CA, USA), and SmartTrack (Align Technology, San Jose, CA, USA) [23]. Each material exhibited a slight level of cytotoxicity that was comparable to other dental materials, e.g., miniscrews or bonding materials. The same study similarly assessed the thermoforming effect on the cytotoxicity of three selected materials (Duran, Biolon, and Zendura). The thermoformed materials showed more cytotoxicity, most likely due to the release of monomers in relation to increasing temperature in the thermoplastic process. An alternative to the described conventional fabrication leading to the fourth revolution in orthodontics—the 3D direct printed clear aligners [24] using dedicated resins (Figure 2).

### 2.3. Development of Clear Aligners- 3D Printing

As previously described—just as the move from orthodontic rings to brackets was made possible through advances in dental materials; the use of clear aligners will spur the research and development of new materials specifically for use in direct 3D printing. 3D printing, also known as additive manufacturing, dates back to the 1980s and is now reaching a level of maturity where it is increasingly being utilized in the fields of dental and medical modeling. There are multiple applications in oral surgery, prosthodontics, restorative dentistry, orthodontics, implantology and instrument manufacturing [24]. 3D printing enables the manufacturing of pieces layer-by-layer instead of common manufacturing methods that rely on machining, molding, and subtractive methods [25]. Current materials used for 3D printing in orthodontics include acrylonitrile-butadiene-styrene plastic, stereolithography materials (epoxy resins), polylactic acid, polyamide (nylon), glass-filled polyamide, silver, steel, titanium, photopolymers, wax, and polycarbonate [26].

An example of the application of 3D printing in the production of clear aligners is the above-mentioned 3D printing of dental models for the clear aligners molding. The use of 3D printed models was the first step towards minimization of errors and mistakes (e.g., geometric inaccuracies) occurring during impression collection [27]. Rather than using error-prone plaster models that are scanned and modeled to develop various alignment stages, it is superior to use digital impression taking and 3D printing for improvement. Use of a clear aligner that is 3D printed for direct usage can eliminate the cumulative errors introduced from analog impression taking and the subsequent thermoplastic workflow [27]. In addition to greater accuracy, direct printing produces other benefits such as shorter supply chains, significantly shorter lead times, and lower costs. It is also a more sustainable process that generates significantly less waste than subtractive and thermoforming processes [28].

3D printing could be also used for direct printing of clear aligners by a single processing step using one or a combination of 3D-printing processes [24]. Despite the current improvements and increased use of different 3D printing technologies, there is a limited number of publications can be found describing the direct 3D printing of orthodontic clear aligners, or research regarding suitable materials for such printing.

Theoretically, various 3D printing processes may be used for direct printed clear aligners, such as fused filament fabrication (FFF), selective laser sintering (SLS) or melting (SLM), stereolithography (SLA), multi-jet photocured polymer process, HP MultiJet Fusion technology or continuous liquid interface production technology [24]. However, due to specific characteristics of clear aligners and specific requirements on their material properties and performance, 3D printing by photo-polymerization from clear resin seems like the most appropriate option.

While there are a number of registered patents describing this possibility, there are only a few recently published articles [24,27,29] that have appeared with the first companies focusing on directly printed aligners and materials for 3D printing. A study conducted by Jindal et al. [27] reported a successfully 3D-printed 0.75-mm thick clear aligner using Dental LT^®^ (Long Term) clear resin (Form Labs, Somerville, MA, USA) and compared its mechanical and geometrical properties to a conventionally manufactured thermoformed dental clear aligners made of Duran foils. The authors suggested that 3D printed resin-based clear dental aligners are more suitable for patient use as they are geometrically more accurate; besides, they could resist more maximum load with a low displacement and can deform elastically with reversibility for lower displacements. Jindal et al. also reported that LT clear resin has been found to be comparable with Duran and Durasoft (Scheu-Dental GmbH, Iserlohn, Germany) thermoplastic materials under the mechanical stress of non-linear compressive forces equivalent to human bite cycles [30]; this means that 3D direct printing has sufficient mechanical strength to resist external loading without a decrease in clinical performance.

However, Dental LT resin is an approved Class IIa biocompatible material with high resistance to breaking and it is ideal for gnathological splints, dental retainers, and other rigid direct-printed orthodontic appliances as functional ones. Its usage for direct 3D printing of clear aligners has not reported before, and all authors are omitting the main limitation of their studies, or rather the lack of clinical data to evaluate the performance of Dental LT resin and its resistance during its use in the oral cavity of patients [31].

Increasingly, a number of preliminary studies regarding 3D printed clear aligners have been carried out in the later stages. As previously mentioned in regards to thermoformed aligners, the thickness of the aligner influences the magnitude of force it delivers to generate optimal tooth movement and performance. 3D printing represents a complex method in controlling the thickness of dental aligners and subsequently the exerted force. Edelmann et al. reported an increase of overall thickness than the corresponding design file, especially with LT clear resins [32]. The results obtained in the study showed that the 3D printing workflow did not precisely realize the designed thickness with clear material and it may produce deleterious effects of clinical utility of aligners.

Deviation in the dimensional accuracy of the printed aligners can result in undesired tooth movement. One of the factors responsible for the adaptation between the aligner and the teeth is the print orientation and the post-print curing duration. McCarty et al. showed little effect on the overall dimensional accuracy of 3D printed aligner design could be also modified in the first stages of printing such as in post-curing of biocompatible resins [33]. Also, Jindal et al. reported that the effects of post-curing conditions on the mechanical characteristics of printed aligners demonstrated that time and temperature are extremely important in the compression load resistance of the printed resin [34]. The correct post-curing process is essential to provide sufficient strength and biocompatibility for the final 3D printed product. Allowing for extra cross linking of the polymer, ultimately enhances mechanical properties of the printed material and reduces any residual stress.

Additive processes, as opposed to subtractive ones, involve the building of parts layer by layer of a liquid photopolymer resin to create a solid polymer. An intrinsic property of 3D layering is mechanical anisotropy; the environmental variations, post-cure time, and bath variations influence the mechanical properties of printed parts. Shanmugasundaram et al. analyzed the mechanical anisotropy in additively manufacturing by stereolithography [35]. It has been shown that printed parts that can be classified as isotropic have a great advantage over other techniques providing for greater accuracy and clinical performances. It was also confirmed that aging and post-cure time had a direct relationship with superior mechanical properties compared to uncured resins and thermoformed clear aligners [27].

3D printed materials are highly toxic before 3D printing, and the toxicity gradually decreases post-polymerization. Post curing and processing are vital for eliminating the toxicity levels as recommended by the manufacture of 3D printing material [36]. The cytotoxicity of directly 3D printed clear aligners from three different resins has been investigated [29]. Three different materials were compared: Accura 60^®^ SLA (3D Systems, Rockhill, SC, USA), Dental LT^®^ clear resin (Form Labs, Somerville, MA, USA), and Invisalign^®^ (Align Technology, San Jose, CA, USA). Similarly, Dental LT resin and Accura 60^®^ SLA are not approved for direct printed clear aligners and were not previously tested nor described for this intention. The study verified that Invisalign^®^ material was the least cytotoxic, followed by Dental LT^®^ and Accura 60^®^. However, Invisalign^®^ aligners are not directly 3D printed. Unfortunately, the absence of clinical data is similarly the main limitation of the study. Recently, an in-vitro study of cell viability by Ahamed et al. showed that Invisalign^®^ material was found to be more biocompatible compared to directly printed aligner materials [36]. Cytotoxicity was found to be more on the first day and then gradually decreases; these results suggest new future studies to evaluate the 3D printed aligners clinically, determining their properties on patients.

Technical properties and cytotoxicity parameters of the most used thermoplastic materials and printing resins are summarized in Table 1 [19,23,29,36].

### 2.4. Materials for 3D Printed Aligners

**Figure 3:**
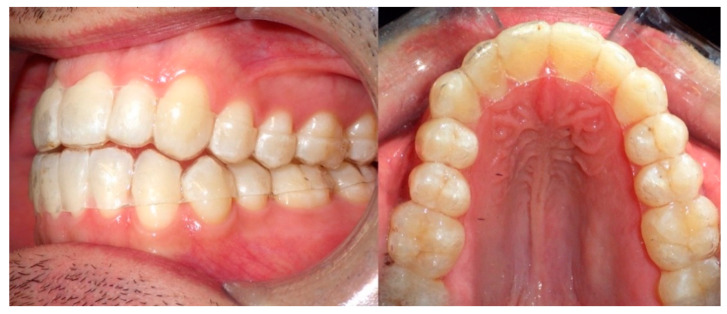
Final step of an orthodontic treatment with 3D direct printed clear aligners (experimental trial on a voluntary patient).

There are multiple clear resins used for 3D printing of appliances in dentistry, but no clear resin is aimed for 3D printed clear aligners. In early 2018 EnvisionTEC (EnvisionTEC Inc., Dearborn, MI, USA) announced the commercial release of E-Ortholign, an innovative material for the direct 3D printing of clear aligners [37]. However, this clear resin is stated as a biocompatible, dimensionally stable, flexible, and strong material for the direct 3D printing of what is known in orthodontics as a “first aligner”—a retainer that is used after an orthodontist removes a patient’s brackets to hold teeth in place before the treatment is completed with a series of conventionally made thermoformed aligners [37]. Nowadays, there is no marketed and approved photopolymerizable resin suited for the direct printing of dental clear aligners but an increasing interest in the field of orthodontics is taking place, especially for the development of certified biocompatible resins [38]. Despite the lack of existing biocompatible resins for 3D direct clear aligners, some experimental trials have been carried out on voluntary patients with modified resins and post-printing protocols, but clinical outcomes need to be confirmed prior to scientific publishing (Figure 3).

**Figure 4:**
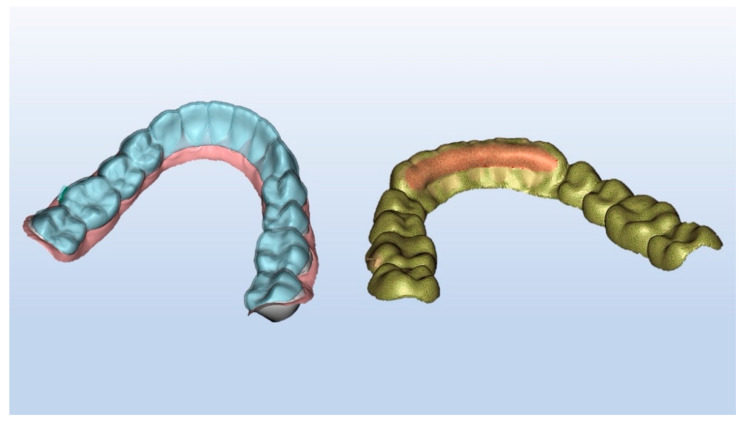
Customization of design and thickness of directly printed aligners.

Likewise, to our knowledge, while there are several CAD software packages for treatment planning and preparation of 3D models for aligner molding, only Maestro 3D (Ortho Studio v.5.2, AGE Solutions S.r.l., Pontedera, Italy) has an add-on module that can also design aligners and export them in a format convenient for direct 3D printing. Thanks to post-export CAD software, it is also possible to fully customize the thickness of direct printed clear aligners in order to exert selective and directional forces (Figure 4).

Objectively, the direct 3D printing of aligners offers several advantages over conventional fabrication, just utilizing the same digital features currently available for construction of bite splints:

- borders are digitally designed and identically reproduced for all sets of aligners;
- edges are smooth and do not need trimming or polishing;
- undercuts do not exist because they are digitally defined;
- aligners are fabricated with higher precision as there are no errors introduced during printing of a 3D molding model and thermoforming stage of fabrication;
- higher precision leads to better fitting and higher effectiveness;
- intra-aligner thickness is customizable, and this may reduce the need for attachments, which generally lower the transparency of clear aligners.

Given these strengths, once an ad-hoc resin is available in the market, directly 3D-printed aligners must be tested clinically to demonstrate their biomechanical effectiveness. Different designs should be probably used for the manufacturing of direct printed clear aligners compared to thermoformed ones due to the possibility of creating customizable thickness and attachments; mechanical properties of customizable aligners regarding the materials, thickness, and auxiliary elements can also be investigated by parametric modeling and finite element analysis [39].

## 3. Conclusions

Three-dimensional printing is used in multiple fields of dentistry. 3D printing technology is a suitable method of fabrication of clear aligners and offers several advantages over the conventional thermoforming process. While the current state of published literature about directly printed clear aligners shows that such a process is technically possible, no approved material marketed for this purpose exists, and software tailored to this aim has to be developed. For this reason, no clinical studies of such printed aligners can be found. Based on these premises, further in vitro and in vivo studies are needed to test these new technologies and materials. Special care must be taken concerning the cytotoxicity of printable resins and clinical performances of direct clear aligners must be calculated to compare the obtained data with those of thermoformed aligners. Orthodontics has definitely entered into a new additive era.

## Figures and Tables

**Table 1:** Technical data (supplier specifications) and cytotoxicity (experimental outcomes) of the most common materials used for thermoformed (T) and directly printed (P) clear aligners.

Brand	Material Composition	Density (g/cm^3^)	Elastic Modulus (MPa)	Tensile Stress (MPa)	Elongation at Break (%)	Cell Viability (%)	Cytotoxicity
**Duran (Scheu-Dental GmbH)** **(T)**	Polyethylene terephthalate glycol (PET-G)	1.27	2200	53	>40	84.6 (Day 14)	Slight
**Biolon** **(Dreve Dentamid GmbH)** **(T)**	Polyethylene terephthalate glycol (PET-G)	1.27	2020	45	>35	64.6 (Day 14)	Slight
**Zendura** **(Bay Material LLC)** **(T)**	Polyurethane resin (PU)	1.203	-	-	-	74.4 (Day 14)	Slight
**SmartTrack (Align Technology)** **(T)**	Multilayer aromatic thermoplastic polyurethane/copolyester	-	-	-	-	94.1 (Day 7) 78.8 (Day 14)	Slight
**Dental LT (Formlabs Inc.)** **(P)**	Photo polymeric clear methacrylate oligomer and glycol methacrylate	1.1 1.3	2300	84	12	60.3 (Day 1) 62.5 (Day 14)	Slight
**Accura 60 SLA (3D System) (P)**	Polycarbonate-based photo-polymeric resin	1.13 1.21	2690–3100	58–68	5–13	13.9 (Day 1) 24.5 (Day 7)	Severe
**E-Guard** **(EnvisionTEC) (P)**	Photo-polymeric clear resin	1.06 1.12	2050–2130	79–85	3.81	64.1 (Day 1) 75.1 (Day 7)	Slight

## Data Availability

No new data were created or analyzed in this study. Data sharing is not applicable to this article.
